# Mediating effect of successful aging on the relationship between psychological resilience and death anxiety among middle-aged and older adults with hypertension

**DOI:** 10.3389/fpubh.2023.1116263

**Published:** 2023-09-19

**Authors:** Meiding Wang, Lin Zhang, Jianing Ma, Hong Sun, Ziyun Gao, Mengya Hu, Haiyang Liu, Leilei Guo

**Affiliations:** ^1^School of Nursing, Jinzhou Medical University, Jinzhou, Liaoning, China; ^2^Department of Internal Medicine Nursing, School of Nursing, Wannan Medical College, Wuhu, Anhui, China; ^3^Student Health Center, Wannan Medical College, Wuhu, Anhui, China

**Keywords:** psychological resilience, successful aging, death anxiety, middle-aged and older adults, hypertension

## Abstract

**Objective:**

The aging trend of China's population is severe and successful aging (SA) is imminent. Aging can lead to various chronic diseases, with hypertension being the most common. Due to this lifelong disease, patients suffer from many anxieties, as death anxiety (DA) can be the most prevalent. Studies have exhibited that middle-aged adults approaching the transition to an older state show more pronounced DA than the more senior. It has been suggested that psychological resilience (PR) can reduce DA. Therefore, this study aimed to analyze the mediating effect of SA between PR and DA in middle-aged and older adults with hypertension.

**Methods:**

A cross-sectional survey was designed. From August to December 2021, 298 middle-aged and older adults with hypertension were selected by multistage cluster random sampling in three districts (Ling he District, Gu ta District, and Tai He District) of Jinzhou City, Liaoning Province. They were surveyed using the demographic questionnaires, the Conner-Davidson Resilience Scale, the Successful Aging Inventory, and the Chinese version of a Likert-type Templer-Death Anxiety Scale. Descriptive analyses, independent sample *T*-test, and one-way analysis of variance (ANOVA) were used to describe demographic characteristics among hypertensive patients with different characteristics, respectively. Statistics were considered significant when *P* < 0.05. Pearson correlation coefficients describe the relationship between PR, SA, and DA. The research model was shaped through Structural Equation Modeling (SEM). SPSS PROCESS macro was used to verify the mediation model. A binary logistic regression model was used with DA as the dependent variable.

**Results:**

The scores for PR, SA, and DA in hypertensive patients are (49.52 ± 14.38) points, (51.22 ± 7.63) points, and (46.67 ± 9.03) points. PR was negatively correlated with DA (*r* = −0.307, *P* < 0.01). Moreover, incorporating SA as a mediating variable in PR and DA, SA was positively correlated with PR (*r* = 0.335, *P* < 0.01) and DA (*r* = 0.085, *P* > 0.05). The direct effect is opposite to the sign of the indirect effect. There is a suppression between PR and DA with a percentage of 20.7%. Good self-assessed health status [0.057 (0.018, 0.183)] may be a protective factor for DA.

**Conclusion:**

Healthcare providers should improve the PR of middle-aged and older adults with hypertension through interventions that reduce DA and increase the likelihood of SA.

## 1. Introduction

China, the second-largest economy in the world, is quickly becoming an aging country (1). China is a country with not only the world's largest population but also the largest aging population; by 2050, the proportion of older people within the total national population is projected to be around 25% (2, 3). Accelerating population aging reports significant impacts on human wellbeing (4). Successful aging (SA) may answer the significant challenges that population aging poses to the health care system, financial security, and labor supply (5). So, SA is an indispensable part of development programs (6). SA is not just about extending life but more about increased awareness, active participation in society, and a proper perspective on aging. Numerous academics have defined SA. Rowe JW and Khan RL proposed the most influential SA concept in 1987, including three major components: low probability of disease and disease-related disability, high cognitive and physical functional capacity, and active engagement with life (7). In subsequent studies, Crowther et al. (8) thought that this concept missed an important factor-positive spirituality and linked positivity with health. From then on, the definition of SA was expanded to four aspects. SA not only leads to longer life spans compared to normal aging but also improves people's quality of life through the positive effects of physical and psychological factors. Frailty and SA represent the opposite sides of the same coin (5). Improving the SA status of the older adults and transforming the adverse effects of aging on society into positive factors are the current focused topics in the field of aging research.

Aging is the primary driver of most chronic diseases (9). Chronic disease can lead to estimated 80% of deaths in China (10), with hypertension leading the way (11). Untreated and improperly managed hypertension can hasten the disease's progression, resulting in serious organ problems and possibly raising the risk of death (12–14). Patients typically die from hypertension's devastating consequences, such as strokes and heart diseases, rather than the condition itself. It is difficult to avoid death anxiety in patients suffering from hypertension. Among these anxiety, death anxiety (DA) is one of the most common. Death is described as an extraordinary fear of death, accompanied by feelings of dread or anxiety when a person considers the process of dying or what happens after death (15). DA is a persistent, abnormal, pathological fear of death. It is not an innate or inherent quality; instead, it is an acquired trait that is impacted by the immediate culture and context (16). It is found that hypertension is the most common complication and risk factor among patients with coronavirus disease 2019 (COVID-19) (17). DA increased significantly during the COVID-19 pandemic (18, 19). Since the beginning of the epidemic, people's lives have been turned upside down. “Death” was one of the most searched terms in the world during the year, it has become commonplace. Delayed access to care and drug scarcity has created panic among the sick, and their awareness and exploration of death have never been that profound. DA refers to anxiety experienced in daily life instead of how a person feels in an emergency, where fear is the feeling that life will end as opposed to the belief that life will go on (20). Throughout the life cycle, we tend to think about how much longer we can exist as independently conscious living beings. Considering the shortening of life span and the approach to death, it is inevitable that people suffer from DA. Guiding patients to express their views on death can help them reduce their anxiety (21). Death is an inevitable outcome of life's journey, and we live in an environment of death denial that leads many people to have more profound anxiety about it. Iverach et al. (22) argues that DA may become lingering stress that endangers personal health. Various factors affect DA, such as age, physical health, and quality of life (23). Research has been done to show that middle-aged adults have higher DA levels than older adults (24). Middle-aged people face the first signs of the aging process. They believe they are so old that they cannot accomplish things well and are gradually being abandoned by society. In the long run, they will develop severe anxiety and depression, also a negative attitude toward aging, and see it as an obstacle to achieving their goals. Therefore, it makes sense that beliefs about aging and old age are thought to affect DA (25).

Previous studies have demonstrated that improving psychological resilience (PR) helps reduce the patient's DA (26). PR, which is defined as the positive function of individual differences in people's response to stress and adversity, was proposed in 1987 by Rutter (27). PR may directly affect an individual's emotions or affect the individual's feelings indirectly by responding. In today's emotional life, DA is anticipated to be the most efficacious unconscious psychodynamic dynamism (28). Higher DA has also been associated with poorer overall mental health and co-occurring mental health conditions (22). DA increases with declining mental health, making it imperative to improve PR.

Current studies have explored the relationship between PR and DA (29), but there are few studies on the relationship between PR, SA, and DA in middle-aged and older adults with hypertension. Therefore, the purpose of this study is to explore the relationship between PR, SA, and DA in hypertensive patients, to provide a reference for formulating effective intervention measures to reduce the level of DA in hypertensive patients.

## 2. Methods

### 2.1. Aim

This study aimed to investigate the mediating effect of SA between PR and DA in middle-aged and older adults with hypertension after considering confounding factors. We hypothesized that SA plays a role between PR and DA in middle-aged and older hypertensive patients.

### 2.2. Study design

A cross-sectional survey of middle-aged and older adults with hypertension was conducted in three districts (Ling he District, Gu ta District, and Tai he District) of Jinzhou City, Liaoning Province, China, from August to December 2021.

### 2.3. Participants

**Figure 1:**
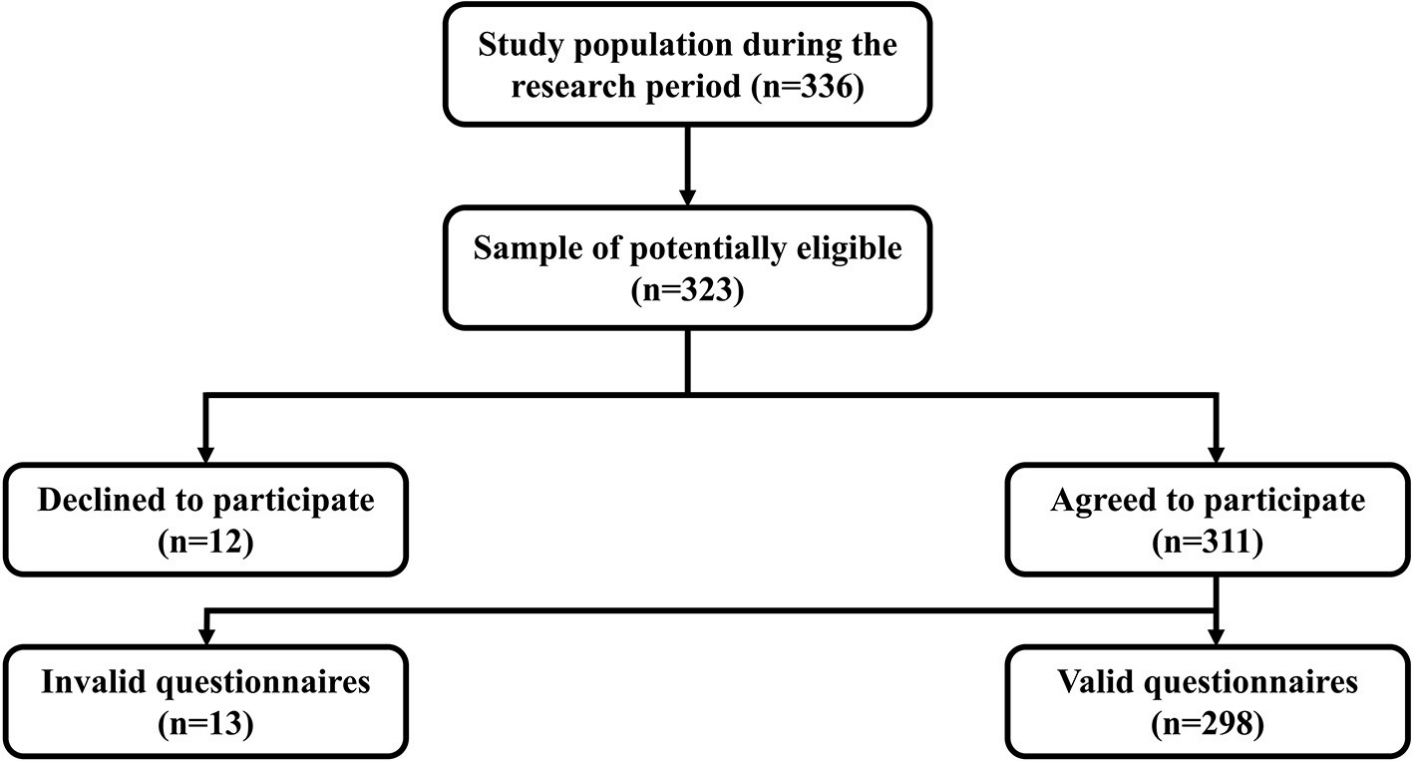
Population flow diagram.

A multistage cluster random sampling was used to conduct this study. In the first stage, two streets in each of the 3 districts (Ling he District, Gu ta District, and Tai he District) were randomly selected as primary sampling units (PSU). Then, two community was randomly selected from each primary sampling unit (PSU) as a secondary sampling unit (SSU). In the third stage, one community health service center was randomly selected from each secondary sampling unit (SSU) to set up a survey site, and 28 middle-aged and older adults with hypertension were randomly selected from each. Finally, 336 middle-aged and older adults with hypertension who met the inclusion criteria were surveyed. To guarantee the required sample size, 311 questionnaires were distributed in this study. Two hundred ninety-eight valid questionnaires were recovered, with an effective rate of 95.8% (Figure 1). The following inclusion criteria were used to choose the participants: (I) diagnosed as hypertension by clinical and pathological diagnosis; (II) conscious, able to communicate normally, and understand the survey content accurately; (III) aged ≥45. Exclusion criteria: Combined with other life-threatening severe diseases.

### 2.4. Study instruments

The questionnaire series consists of demographic questionnaires (i.e., gender, age, educational level, et al.) and three well-established instruments for collecting data on PR, SA, and DA.

#### 2.4.1. Socio-demographic characteristics questionnaire

After a comprehensive literature review and rigorous group discussions, a questionnaire on socio-demographic characteristics, including gender, age, education level, place of residence, health behavior (smoking habits, alcohol habits, and physical activity), and self-assessed health status was finally developed.

#### 2.4.2. Psychological resilience

Psychological resilience was assessed with the 25 items Conner-Davidson Resilience Scale (CD-RISC) was compiled by Connor and Davidson (30), which includes five dimensions, including “competency and tenacity,” “tolerance of negative affect,” “positive acceptance of change,” “control,” and “spiritual influences.” The scale uses a 5-point Likert-type scale with the values 0 (strongly disagree) to 4 (strongly agree) to rate a person's psychological resilience. The total score ranges between 0 and 100, and the score positively correlates with the degree of PR felt by individuals; that is, the higher the score, the better the psychological resilience. The Cronbach's Alpha for the CD-RISC was 0.865 in the current study.

#### 2.4.3. Successful aging

The Successful Aging Inventory (SAI) was compiled by Troutman et al. (31). This study takes a brief version of successful aging by Lee et al. (32), which is valid and reliable for detecting SA. Such a scale is made up of 16 items and four dimensions, including “proactive engagement,” “wellness resources,” “positive spirit,” and “valued relationships,” the answers to each item encompassed four levels, including not at all, sometimes, usually, and critical, for which scores were 1, 2, 3, and 4, respectively. The total score ranges from 16 to 64 points. Greater scores indicate better SA. The Cronbach's α-value of the scale was 0.862 in this study.

#### 2.4.4. Death anxiety

The Templer's Death Anxiety Scale (T-DAS) was developed by Templer (33). This study adopts the Chinese version translated and revised by Yang et al. (34) composed of 15 items, namely the Chinese version of the Templer death anxiety scale (CT-DAS). The CT-DAS consists of 15 questions with a true or false answer. A 5-point Likert-type of CT-DAS was evaluated and validated in patients with colorectal cancer. The scale includes 15 items and four dimensions, including “Emotion,” “stress and pain,” “Time consciousness,” and “Cognition,” scoring on a 5-point Likert-type scale ranging from 1 (strongly disagree) to 5 (strongly agree). Among them, nine items are positive scoring; in contrast, the other six items are scored in reverse. The total score on the Likert scale can range from 15 to 75. The higher the score, the higher the individuals' DA level. The Cronbach's Alpha for the CL-TDAS was 0.798 in the current study.

### 2.5. Covariates

Age, gender, and self-assessed health status were used as covariates in this study. Gender is divided into male and female. Self-assessed health status includes poor, common, and good.

### 2.6. Data collection

To reduce errors, relevant research staff were trained before the survey, and uniform instructional language was used to clarify the purpose of the survey and scoring criteria. Before this interview, each participant was told that the survey was anonymous and credible to eliminate the concerns of patients and answer truthfully. Informed consent forms were signed, and paper questionnaires were distributed to patients who met the inclusion criteria after explaining the survey's purpose and questionnaire requirements. The subjects answered the questionnaires themselves. The questionnaires were returned on the spot after completion. The investigators asked the subjects face-to-face to illiterate adults and then filled in the questionnaires.

Sample sizes were determined to be 5–10 times the number of scale items with the most items using Kendall's sample calculation method (35). In this study, the scale with the highest number of entries was the Conner-Davidson Resilience Scale, which consisted of 25. Also, a 20% sample loss was considered, so the sample size for this study was at least 150–300. To secure the sample size requirements, 311 questionnaires were disseminated in this study. Two hundred ninety-eight valid questionnaires were recovered, with an effective rate of 95.8%.

### 2.7. Data analysis

The SPSS 26.0 software was used for data input and analysis. Descriptive analyses, independent sample *T*-test, and one-way analysis of variance (ANOVA) were used to describe demographic characteristics among hypertensive patients with different characteristics, respectively. Binary variables such as gender were compared by independent samples *t*-test; polytomous variables such as education levels and place of residence were compared by one-way analysis of variance (ANOVA). *P* < 0.05 was considered statistically significant. Pearson correlation coefficients described the relationship between the three variables (PR, SA, DA). Furthermore, a mediation model with PR as the independent variable, SA as the mediating variable, and DA as the dependent variable was set up. The SPSS PROCESS 3.5 macro (Model 4) was utilized to examine the mediating function of SA (36). Ninety-five percent confidence intervals (CIs) were calculated using the bias-corrected non-parametric percentile bootstrapping method with 5,000 samples. If the confidence interval includes zero, it means that there is no significant mediating (indirect) effect at the significance level of 5%.

### 2.8. Ethical consideration

All individuals have provided informed consent before the data collection. Approval for this study was given by the Medical Ethics Committee of Wannan Medical College (approval number: 2021-3), and all participants provided informed consent. All methods were performed in accordance with the Declaration of Helsinki.

## 3. Results

### 3.1. Descriptive statistic

Table 1 shows the demographic characteristics of the 298 study objects. Among them, 190 (63.8%) were male, and 108 (36.2%) were female. The age range of the middle-aged and older adults with hypertension was 45–96 years. Most of the study participants (69.4%) reported Junior high school education or less. The proportion of patients living in city and Countryside is basically the same. The number of non-smokers and non-drinkers was both over half. Only 10.4% of the patients considered themselves in good health.

**Table 1:** Demographic characteristics of middle-aged and older adults with hypertension (*N* = 298).

**Characteristic**		**Frequency (*n*)**	**Rate (%)**
Gender	Male	190	63.8
	Female	108	36.2
Age (years)	45–54	59	19.8
	55–64	90	30.2
	65–74	109	36.6
	75–84	33	11.1
	≥85	7	2.3
Educational level	Primary school or less	116	38.9
	Junior high school	91	30.5
	Senior high school or above	91	30.5
Place of residence	City	144	48.3
	Suburban	31	10.4
	Countryside	123	41.3
Smoking habits	No	160	53.7
	Ever smoke	99	33.2
	Current smoke	39	13.1
Alcohol habits	No	167	56.0
	Ever drink	62	20.8
	Current drink	69	23.2
Physical activity	No	48	16.1
	Irregular physical activity	175	58.7
	Regular physical activity	75	25.2
Self-assessed health status	Poor	134	45.0
	Common	133	44.6
	Good	31	10.4

### 3.2. The scores of psychological resilience, successful aging, and death anxiety with different characteristics

Table 2 demonstrates the comparison of PR, SA, and DA scores of hypertensive patients with different characteristics. The scores for PR, SA, and DA in hypertensive patients are (49.52 ± 14.38) points (51.22 ± 7.63) points, and (46.67 ± 9.03) points. Education level, alcohol habits, physical activity, and self-assessed health status were factors that significantly affected the PR of hypertensive patients (*P* < 0.05). Age, place of residence, and self-assessed health status are factors that significantly influence SA in hypertensive patients (*P* < 0.05). There were differences between the DA in physical activity and self-assessed health status (*P* < 0.05).

**Table 2:** Comparison of the score of psychology resilience, successful aging, and death anxiety in different social-demographic subgroups (*N* = 298).

**Variables**	***N***	**CD-RISC**		***F/t***	***P***	**SAI**		***F/t***	***P***	**CL-TDAS**		***F/t***	***P***
		**M**	**SD**			**M**	**SD**			**M**	**SD**		
Gender				1.108	0.269			0.804	0.422			−1.164	0.245
Male	190	50.22	14.44			51.49	7.31			46.21	8.74		
Female	108	48.30	14.27			50.75	8.18			47.48	9.50		
Age (years)				0.496	0.739			2.869	0.023			0.551	0.699
45–54	59	51.29	15.44			50.85	8.54			47.45	8.32		
55–64	90	48.73	15.45			49.38	8.20			45.76	10.13		
65–74	109	49.59	14.23			52.75	6.88			46.55	8.41		
75–84	33	49.33	10.75			52.36	5.43			48.12	9.76		
≥85	7	44.57	8.12			48.86	7.99			46.94	5.84		
Educational level				9.508	< 0.001			1.940	0.145			1.028	0.359
Primary school or less	116	47.46	13.55			51.38	6.72			46.54	9.24		
Junior high school	91	46.84	12.55			50.02	7.76			47.71	9.43		
Senior high school or above	91	54.84	15.79			52.22	8.47			45.81	8.32		
Residence				2.374	0.095			4.116	0.017			0.968	0.381
City	144	51.33	14.50			52.38	7.51			46.55	8.73		
Suburban	31	46.61	16.00			48.55	5.57			48.78	8.66		
Countryside	123	48.13	13.64			50.54	8.01			46.29	9.46		
Smoking habits				0.837	0.434			0.527	0.591			0.850	0.428
No	160	48.52	14.05			50.81	8.16			47.23	9.22		
Ever smoke	99	50.67	15.63			51.78	7.04			46.33	9.00		
Current Smoke	39	50.72	12.34			51.51	6.87			45.26	8.31		
Alcohol habits				3.367	0.036			1.156	0.316			2.866	0.059
No	167	48.44	13.74			50.77	7.68			47.44	9.26		
Ever drink	62	48.10	15.85			51.11	8.90			44.27	8.98		
Current drink	69	53.42	14.02			52.42	6.11			46.97	8.23		
Physical activity				5.871	0.003			0.052	0.949			3.856	0.022
No	48	44.40	13.64			50.90	7.13			47.25	9.46		
Irregular physical activity	175	49.30	14.10			51.29	7.38			47.58	8.90		
Regular physical activity	75	53.32	14.59			51.27	8.55			44.20	8.71		
Self-assessed health status				9.754	< 0.001			3.451	0.033			25.503	< 0.001
Poor	134	45.66	12.65			52.11	7.06			49.45	7.60		
Common	133	52.15	14.95			51.03	8.25			45.96	8.72		
Good	31	54.90	15.12			48.19	6.57			37.74	9.89		

CD-RISC, measurement of psychological resilience; SAI, measurement of successful aging; CL-TDAS: measurement of death anxiety.

### 3.3. Bivariate correlation analyses

Table 3 displays the correlational analysis between the study variables. DA was found to be negatively correlated with PR (*r* = −0.307, *P* < 0.01). SA was positively correlated with PR (*r* = 0.335, *P* < 0.01) and DA (*r* = 0.085, *P* > 0.05).

**Table 3:** Correlation between the study variables (*N* = 298).

	**1**	**2**	**3**	**4**	**5**	**6**	**7**	**8**	**9**	**10**	**11**	**12**	**13**	**14**	**15**	**16**
1. CD-RISC	1	0.928^**^	0.931^**^	0.817^**^	0.862^**^	0.662^**^	0.335^**^	0.381^**^	0.233^**^	0.287^**^	0.218^**^	−0.307^**^	−0.271^**^	−0.253^**^	−0.294^**^	−0.230^**^
2. Compentency and tenacity		1	0.805^**^	0.653^**^	0.780^**^	0.574^**^	0.254^**^	0.297^**^	0.168^**^	0.235^**^	0.145^*^	−0.322^**^	−0.304^**^	−0.250^**^	−0.279^**^	−0.241^**^
3. Tolerance of negative effect			1	0.693^**^	0.793^**^	0.550^**^	0.364^**^	0.424^**^	0.242^**^	0.325^**^	0.222^**^	−0.225^**^	−0.183^**^	−0.198^**^	−0.210^**^	−0.198^**^
4. Positive acceptance of change				1	0.627^**^	0.523^**^	0.296^**^	0.272^**^	0.246^**^	0.210^**^	0.270^**^	−0.258^**^	−0.235^**^	−0.217^**^	−0.286^**^	−0.141^*^
5. Control					1	0.459^**^	0.327^**^	0.383^**^	0.196^**^	0.293^**^	0.214^**^	−0.293^**^	−0.247^**^	−0.248^**^	−0.284^**^	−0.235^**^
6. Spiritual influences						1	0.147^*^	0.228^**^	0.127^*^	0.094	0.044	−0.209^**^	−0.166^**^	−0.171^**^	−0.239^**^	−0.160^**^
7. SAI							1	0.831^**^	0.839^**^	0.885^**^	0.832^**^	0.085	0.062	−0.056	0.087	0.177^**^
8. Proactive engagement								1	0.627^**^	0.651^**^	0.503^**^	−0.023	−0.030	−0.160^**^	0.008	0.079
9. Wellness resources									1	0.659^**^	0.624^**^	0.095	0.058	−0.010	0.103	0.176^**^
10. Positive spirit										1	0.678^**^	0.130^*^	0.102	0.010	0.144^*^	0.178^**^
11. Valued relationships											1	0.098	0.088	−0.015	0.052	0.177^**^
12. CL-TDAS												1	0.936^**^	0.817^**^	0.722^*^	0.859^**^
13. Emotion													1	0.702^**^	0.530^**^	0.729^**^
14. Stress and pain														1	0.523^**^	0.642^**^
15. Time consciousness															1	0.540^**^
16. Cognition																1

CD-RISC, Conner-Davidson resilience scale; SAI, Successful Aging Inventory; CL-TDAS, the 5-point Likert-type CT-DAS.

Competency and tenacity ~ Spiritual influences are items of CD-RISC; Proactive engagement ~ Valued relationships are items of SAI; Emotion ~ Cognition are items of CL-TDAS.

^*^P < 0.05.

^**^P < 0.01.

### 3.4. Model fitness indices

The value of the model fit indicators used in this study is displayed in Table 4. The model fitting results show that, CFI = 0.949; GFI = 0.920; AGFI = 0.880; RMSEA = 0.081, the judgment value is well-fitted, and the model is acceptable.

**Table 4:** The model fit index (*N* = 298).

**Index**	***P***	**CFI**	**GFI**	**AGFI**	**RMSEA**
Change range	-	0–1	0–1	-	-
Reference standard	< 0.05	>0.90	>0.90	>0.90	< 0.080
Actual value	< 0.001	0.949	0.920	0.880	0.081

### 3.5. Relationship between psychological resilience, successful aging, and death anxiety assessed by the SEM

**Figure 2:**
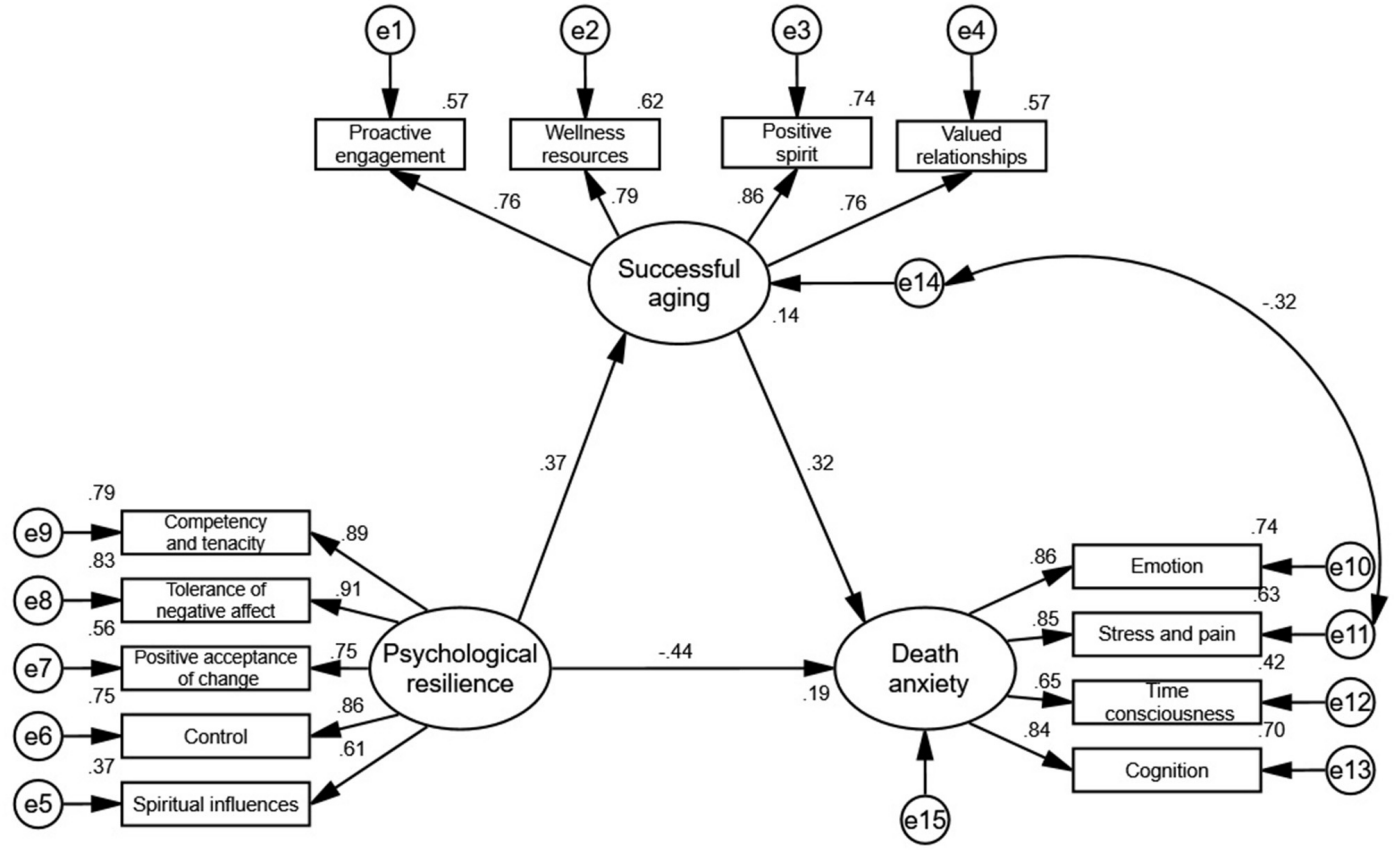
SEM of the mediating role of successful aging between psychological resilience and death anxiety.

The relationship between PR, SA, and DA of the middle-aged and aged patients with hypertension is shown in Figure 2. There were three unobserved variables in this study: PR, SA, and DA. Among them, SA and DA were both represented by four observation variables, and PR was represented by five observation variables.

As illustrated in Figure 2, PR exerted a negative effect on DA (the standardized effect = −0.44, *P* < 0.001). Moreover, PR exerted a positive effect on SA (the standardized effect = 0.37, *P* < 0.001), SA also exerted a positive effect on DA (the standardized effect = 0.32, *P* < 0.001).

### 3.6. The mediating effect of successful aging on the association between psychological resilience and death anxiety

Table 5 shows the standardized total effect, direct effect, indirect effects, as well as the results of the mediating effect. With DA as the dependent variable, SA as the mediating variable, and PR as the independent variable, a structural equation model was constructed. Using the PROCESS 3.3 macro (Model 4) proposed by Hayes (36) to examine the mediating effect of SA in the relationship between PR and DA, the results showed that the Bootstrap 95% confidence intervals for the direct and indirect effects of PR on DA did not include 0. The results showed direct effect [B = −0.179, SE = 0.037, 95% CI = (−0.251, −0.107)] and indirect effects [B = 0.037, SE = 0.017, 95% CI = (0.006, 0.072)] of PR on DA by testing 95% confidence intervals (CIs) based on 5,000 bootstrapped samples, indicating SA partially mediated the relationship between PR and DA. The direct effect is opposite to the sign of the indirect effect. Therefore, SA has a suppression effect rather than a mediating effect between PR and DA with a percentage of |ab/c'| = |0.037/−0.179| = 20.7%.

**Table 5:** The standardized total, direct, and indirect effects of psychological resilience on death anxiety with successful aging as mediators (*N* = 298).

**Path**	**Effect**	**SE**	**95% CI**	
			**LLCL**	**ULCL**
Direct effect	−0.179	0.037	−0.251	−0.107
Psychological resilience → Death anxiety				
Indirect effect	0.037	0.017	0.006	0.072
Psychological resilience → Successful aging				
Successful aging → Death anxiety				
Total effect	−0.142	0.034	−0.210	−0.075

As shown in Table 6, we examined the suppression role of SA on the relationship between PR and DA with the linear regression analysis after controlling for the demographic variables of gender, age, and self-assessed health status. Model 1 showed that PR was negatively associated with DA (B = −0.143, *p* < 0.001), and PR explained the total of 17.7% of the DA (*F* = 16.986, *P* < 0.001). Model 2 showed that PR was positively associated with SA (B = 0.210, *p* < 0.001), and PR explained the total of 16.5% of the SA (*F* = 15.644, *P* < 0.001). Model 3 showed that SA was positively associated with DA (B = 0.172, *p* < 0.001), and the explanation of PR for DA increased from 17.7 to 19.2% (*F* = 15.125, *P* < 0.001).

**Table 6:** Linear regression of hypothesized relationships (*N* = 298).

**Predictors**	**Model 1 (DA)**				**Model 2 (SA)**				**Model 3 (DA)**			
	**B**	**SE**	***T***	**95% CI**	**B**	**SE**	***t***	**95% CI**	**B**	**SE**	***t***	**95% CI**
Gender	1.128	0.997	1.132	−0.834, 3.090	−0.057	0.849	−0.067	−1.727, 1.614	1.138	0.988	1.152	−0.806, 3.082
Age	−0.036	0.477	−0.075	−0.975, 0.903	0.775	0.406	1.908	−0.024, 1.575	−0.169	0.476	−0.356	−1.106, 0.767
Self–assessed health status	−4.280	0.743	−5.759^***^	−5.743, −2.818	−2.700	0.633	−4.267^***^	−3.946, −1.455	−3.815	0.759	−5.026^***^	−5.309, −2.321
PR	−0.143	0.034	−4.171^***^	−0.210, −0.075	0.210	0.029	7.228^***^	0.153, 0.268	−0.179	0.037	−4.863^***^	−0.251, −0.106
SA									0.172	0.068	2.534^***^	0.039, 0.306
Adjusted *R^2^*	0.177				0.165				0.192			
*F*	16.986^***^				15.644^***^				15.125^***^			

^***^P < 0.001.

### 3.7. Logistic regression analysis with death anxiety screening results as the dependent variable

**Figure 3:**
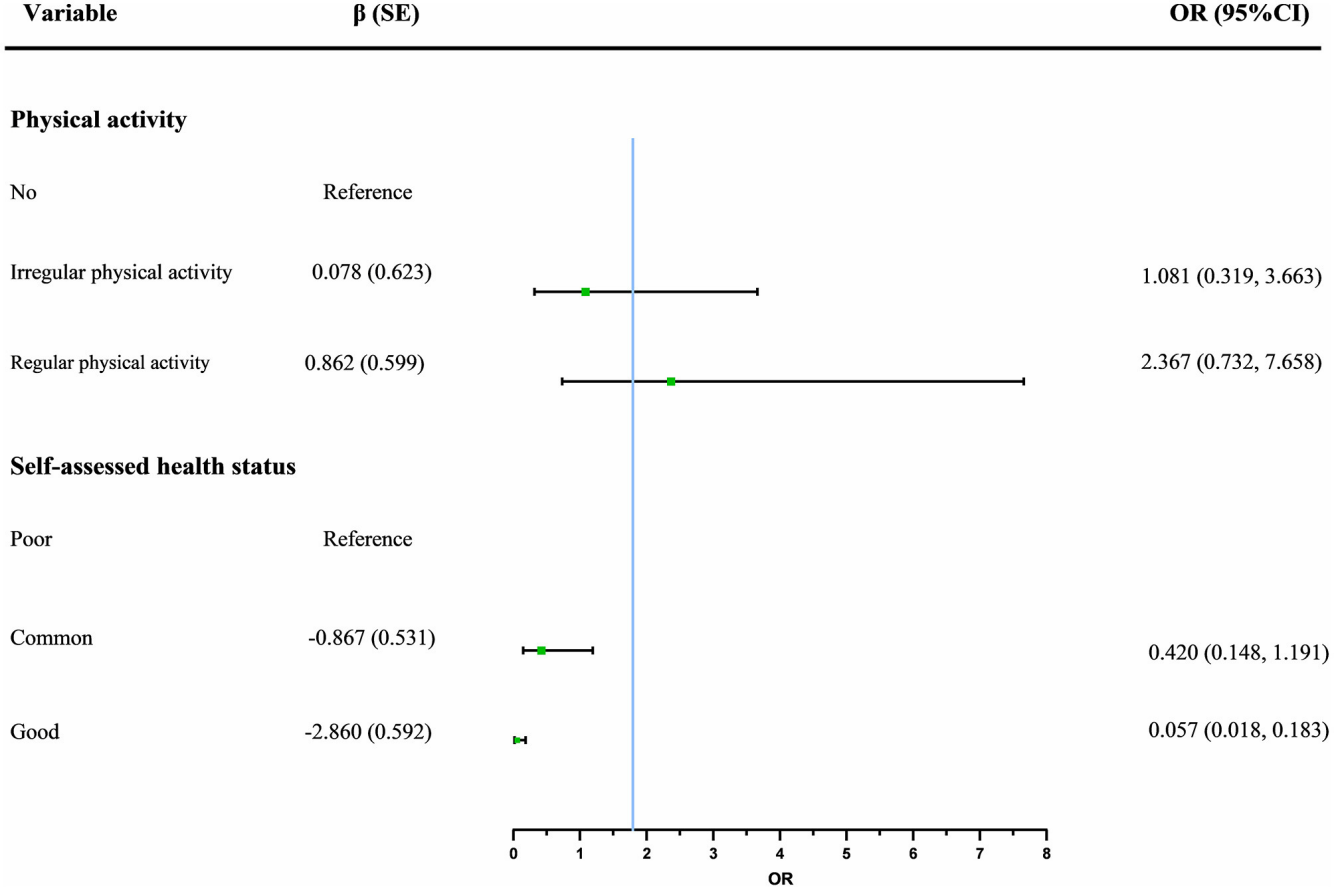
Effects of different types of physical activity and self-assessed health status on death anxiety (using the low level and no exercise as the reference).

A binary logistic regression was performed with DA as the dependent variable and statistically significant in the univariate analysis as the independent variable. DA was defined as a cut-off score of 35, with a score below 35 representing low DA and a score ≥35 representing high DA. Good self-assessed health status (*P* < 0.05) was associated with low DA, and the higher the level, the more significant the protective effect (Figure 3).

## 4. Discussion

The study, conducted in three districts in Jinzhou, Liaoning Province, constructs a mediating model to explore whether PR is indirectly associated with DA through SA. The results suggest that the effect of PR on DA can be partially explained by SA. This study is the first to explore the mediating effect of SA and provides a theoretical basis and direction for reducing DA and increase the possibility of SA in middle-aged and aged hypertensive patients.

### 4.1. Death anxiety of middle-aged and older adults with hypertension

Older and middle-aged hypertensive patients in this study have a relatively high state of DA, which is slightly higher than that in previous studies (37). The reasons why they are prone to DA include the following. Firstly, most patients are unaware of the process and improvement options for the onset and progression of hypertension. It must be recognized that several factors, including economic status and a lack of resources, make it difficult for Chinese patients with hypertension to obtain health information from healthcare providers (38, 39). The high risk of complications from poorly controlled hypertension is one of the leading causes of anxiety and dread among middle-aged and older adults with hypertension, in addition to the fear of the likelihood of getting various diseases rising with age. A person's health depends on access to and utilizing public health information. Healthcare providers ought to utilize both online and offline tools to broadly disseminate health information and emphasize the importance of timely blood pressure regulation in order to increase public awareness of hypertension. Secondly, the process of hypertension is irreversible and progressive. The disease always chronically afflicts the patients. The inevitability of death is reminded almost every day, which makes them feel the threat of death deep inside. Thirdly, poor health is a significant factor influencing DA in this study, mainly because the changes in lifestyle and the lifelong medication disrupt the life balance of the patients.

Lonette classifies four common patterns of DA as the following: (a) cognitive-emotional problems, (b) concerns about physical changes, (c) concerns about the passage of time, and (d) concerns about stressors and pain concerns (40). The fear of death and the uncertainty about what would happen after death seems to be essential parts of the human mind. Previous studies have proven that physical health was negatively associated with DA (41). It means that the worse the physical condition is, the higher the level of DA is. When the physical state and the quality of the life are seriously reduced, the patients will feel no dignity, which undoubtedly causes a significant blow to the patient, seriously affects the patient's psychological state, and even aggravates the patient's psychological burden. DA can create negative emotions that are detrimental to the treatment of the disease and effective communication between the doctor and the patient. Furthermore, it may even lead to more serious psychological or physical problems. DA may be closer to a depressive defense mechanism rather than anxiety (42). Like earlier studies (43–45), physical exercise was a key influence in our research. In middle-aged and older adults with hypertension, physical activity reduces high mortality concerns and improves the quality of life, and it is safe and inexpensive. That is possible at any time and wherever. It is a cornerstone medication for preventing and treating hypertension and is considered a psychotropic treatment (46, 47). A considerable risk factor for increased cardiovascular disease mortality has been identified as little physical exercise (48, 49). Regular exercise is one of the most critical aspects in enhancing the existing state of hypertension. It is a good adjunct therapy that can be used in addition to medicine. The guidelines suggest 75 min of vigorous or 150 min of moderate exercise per week (50). However, the fact is that physical activity in hypertensive patients is not ideal, they were less physically taking part than the non-hypertensive individuals (51). Even more, the results from studies have demonstrated that the probability of hypertension increases with age. The risk of getting hypertension rises roughly 20-fold in individuals 65 and older (52). In the long run, a situation like that could lead to a vicious cycle. Therefore, regular physical exercise is more crucial for middle-aged and older adults with hypertension.

Healthcare providers ought to render exercise more enjoyable, encourage patients to work out on schedule, warn them about the seriousness of uncontrolled hypertension, and follow up frequently. Exercise interventions usually require individualized exercise prescriptions by health care providers for each patient. When exercise programs are adequately customized, patients can gain from lower blood pressure, body mechanism regulation, overall quality of life and health improvement. When creating and implementing an exercise program, several safety considerations should be made due to the poor health status of individuals with hypertension. Both the regularity and the intensity of the exercise are essential. Besides, exercise therapy should also be carried out with consideration for the patient's spiritual and psychological state. Low, moderate, vigorous, and strenuous intensity are the broad categories used to classify exercise training. There are plenty of distinct exercise rehabilitation techniques for hypertension. Western doctors frequently use aerobic and anaerobic exercises, while Chinese doctors select exercises that integrate traditional Chinese culture. Usually, aerobic exercise primarily consists of strolling, cycling, and jogging. Dumbbells and weight training are frequently used in anaerobic exercise. Exercise, both aerobic and anaerobic, has been displayed through investigations to substantially lower blood pressure levels in people with hypertension (53–55). Baduanjin exercise and Tai Chi are the two most widely practiced Chinese forms of exercise. Bajuanjin exercise, which derives from the martial arts of ancient Eastern cultures, is a gentle exercise combining breathing and meditative training to improve balance and awaken bodily consciousness. This is why it has always been well-liked. Numerous studies prove that Baduanjin exercise successfully reduces blood pressure (56, 57). Tai Chi has its origins in ancient traditional Chinese martial sports. The activity has been used for both bodily and psychological healing for several generations (58). The kind, as mentioned earlier, of conventional Chinese exercise successfully decreases blood pressure (59). Even patients' quality of life, anxiety, and cognitive function can be enhanced by Tai chi (60). Human beings dramatically benefit from this activity. In addition, in today's technologically advanced age, medical practitioners have developed tools to assist and monitor patients in their exercise (61, 62). These e-health program help patients improve their physical activity, help healthcare providers disseminate health information broadly, and facilitate regular follow-up.

In summary, the effect of exercise on people's health is receiving more and more attention in the structure of physical medicine integration; regular physical activity of any kind can help hypertensive patients recover more quickly by lowering blood pressure and defending target organs.

### 4.2. Psychological resilience and death anxiety

Our study's correlation analysis revealed that PR was negatively associated with DA in middle-aged and older hypertensive patients. In other words, higher PR can help reduce DA in hypertensive patients. This result is in line with findings from earlier research (63). The PR level among hypertensive patients was in the middle level in our study, slightly lower than previous studies of cancer patients (64, 65). The possible explanation is that hypertension is a lifelong disease, and its intractability and recurrence increase the psychological burden of patients, leading to lower PR level. In turn, patients with lower PR are more likely to experience psychological distress (66). Therefore, medical staff should intervene when they detect a decrease in patients' PR levels. Besides, this result also suggests to medical professionals that there is room to improve the current level of PR in middle-aged and older adults with hypertension.

People with high blood pressure are reportedly under tremendous stress, which makes them relatively less resilient (67). PR is a crucial protective factor for patients with hypertension as their condition worsens, which may considerably impact people's ability to adapt to severe sickness (68). Patients with high PR show more significant improvements in social functioning, mood, and mental health than those with low PR (69). This means that PR is dynamic and can be improved through acquired training. Improving PR can help patients develop or promote resistance to illness and stress, stimulate their potential, and prevent them from developing more serious psychological problems. Attitude toward death affects patients' PR level, and negative death attitudes such as DA are detrimental to older adults' physical and mental health. In addition, family as an essential social component has a positive effect on improving PR and reducing DA. A good family atmosphere, care, and support from family members can provide mental state support for middle-aged and older adults with hypertension. Educational attainment is a significant factor influencing PR in our study, consistent with previous findings (70). Higher educational attainment somehow increases patients' perceptions. Patients with higher levels of education may be more likely to access relevant information about hypertensive disorders through various sources. As a result, they better understand the disease and gain greater control over the treatment process. Therefore, when DA is prevalent in hypertensive patients, health care professionals should activate protective mechanisms for their PR and develop appropriate interventions to reduce DA in patients.

### 4.3. Psychological resilience and successful aging

This study shows a significant positive relationship between PR and SA. Such a result illustrates that PR can effectively mitigate mental deterioration. When confronted with aging difficulties, individuals with high resilience generally redirect their attention to what they can still do rather than what they cannot (71). This may be because they have summed up a lot of life experience from past setbacks and tribulations and are able to adjust their mindset and maintain a stable emotion in a timely and effective manner. It is crucial to distinguish PR from physical resilience (health) because many adults will encounter health issues (72). Health status is the most critical factor affecting SA (73). Health status includes physical health and mental health. Most traditional definitions of SA are based on the absence of somatic disorders and less often include psychological factors (74). However, psychological factors are an essential part of achieving SA. It is thought to be significantly related to SA (75). SA maximizes one's psychological resources, namely self-efficacy and resilience (76). In situations involving anxiety and the psychological issues brought on by long-term conditions, PR reduces the detrimental impacts of adversity on mental health (77). High PR can alter how stressful situations are perceived by an individual, which in turn reduces physiological reactions to stressful conditions, especially when reinforced by favorable environmental factors and positive social relationships (78). Although chronic diseases are not easily ameliorated, psychological conditions can be changed through intervention and treatment. Additionally, once sufficiently developed and continuously managed, PR can be long-lasting for a person (79). Therefore, when middle-aged and older adults with hypertension have decreased PR due to aging, medical professionals should promptly correct their perceptions of aging to improve the likelihood of SA.

### 4.4. Successful aging and death anxiety and the suppression effect of successful aging

A situation where the magnitude of the relationship between an independent and dependent variable becomes more prominent when a third variable is included would indicate suppression (80). This study showed a significant negative association between PR and DA. At the same time, when SA was included as a third variable, PR positively influenced DA through SA, suggesting that SA plays a masking role in this study. That is, undesirable SA increases the negative association between PR and DA. It is worth noting that studies show that the SA of our country is not optimistic. This may be due to several reasons. For one thing, aging implies the aging of organs, physical deterioration, and chronic diseases, which are contrary to the concept of SA and are all detrimental to achieving SA. This is precisely why the SA scores of those over 85 years old in this study were the lowest. Also, this study's SA of middle-aged adults was relatively poor. This is because middle-aged people need to face tremendous life stress and feel uneasy and anxious about the impending aging state. In another, place of residence significantly influences SA, which may be due to firstly, the risk of hypertension is more remarkable in rural than in urban areas (81), which may be related to the different dietary habits and living habits between the two regions. Secondly, the healthcare conditions in rural areas are relatively poor. Thirdly, the lack of transportation in rural areas may prevent patients from delaying access to medical care. Fourth, rural patients may have a lower level of education, which leads to a lack of awareness of hypertension and is more prone to panic and possible inappropriate medication use. Fifth, patients in rural areas are relatively less aware of medical examinations. What's more, China's public health system is in its infancy, and its healthcare system and pension mechanism are still underdeveloped. In addition, the late start of compulsory education in China has indirectly led to the low literacy level of some Chinese people and their inability to view aging correctly, leading to the inability to achieve SA. In the end, a taboo culture of death prevails in China, and discussing death is rarely allowed in China. In this case, achieving SA is tough.

In our study, the SA of middle-aged and older hypertensive patients was not ideal. This is because hypertension is an important stress factor. Meanwhile, aging itself is a powerful risk factor for the development of hypertension (82). Patients with hypertension are concerned about their survival time, quality of life, and treatment outcome. However, as a lifelong disease, patients who suffer so much need not only long-term medication, functional exercise, and dietary changes but also frequent hospital checkups, which cause a lot of inconvenience and physical and mental exhaustion to middle-aged and older adults with hypertension. As the old Chinese saying goes, every medicament has its side-effect. The lifelong nature of hypertension treatment with medication will inevitably lead to different degrees of damage to patients' tissues, organs, and body functions. Patients with hypertension suffer from a decline in health, and those with severe conditions even develop related complications, which is hugely detrimental to achieving SA.

Death can happen at any age, as is well-known, yet it has often been accepted that getting older portends death. As people move into old age, their physical functions decline, and various diseases follow. They no longer think death is out of reach. Individuals routinely suffer DA when they recognize their own death (25). Everyone has DA because everyone will eventually experience death. It is a natural and normal feeling that continues throughout our lives. It is rooted in the uncertainty of the afterlife and the painful process of dying. Mild DA is necessary to help us cope positively with what is going on in our lives, but high DA can disrupt people's lives and become a worm in their minds. If DA is ignored, it may lead to an increasing lack of the individual's ability to perceive aging and be far removed from SA. Even though it is well-recognized that death is a remarkable fact of human life, people never feel entirely prepared for such a reality.

Therefore, first, medical staff should conduct regular lectures on hypertension to achieve early screening, diagnosis, and intervention, change their poor lifestyle and dietary habits, develop disease treatment plans, and follow up with them on time. Caregivers can improve patients' PR and reduce disease-related stress through mental health training, information support, and intensive training. Second, healthcare workers should promote proper attitudes toward aging patients; aging is not an irreversible stage of life but an age of infinite possibilities. Again, medical workers and the media should break the traditional culture of keeping their mouths shut about death and educate the public about it. Finally, it is urgent to improve the healthcare system promptly and strengthen the construction and services of aged institutions.

### 4.5. Implications

Recently, SA has become a significant issue due to the increase in the aging population. This study is the first to explore the mediating relationship between SA in PR and DA, which contributes to a deeper understanding of the mechanisms underlying the onset of DA among middle-aged and older adults with hypertension. Practically speaking, this study might offer hypertension patients a new strategy for achieving SA, something the medical staff should take note of. In addition, declining health status due to aging may hurt acquiring SA. We should focus on patients with irreversible diseases and intervene in their mental health to reduce their DA level in time to increase the likelihood of achieving SA.

### 4.6. Limitations

There are some limitations to our research. First, our study was merely cross-sectional; further longitudinal studies are required to ascertain the findings. Second, DA of hypertensive patients is affected by various factors. This study only includes SA as an intermediary variable to explore the relationship between PR and DA among hypertensive patients. In the future, more variables need to be added under the guidance of theory and professionals to explore the mechanism of DA deeply. Third, this study exclusively looked at middle-aged and older hypertension patients in Jinzhou City because of the COVID-19 epidemic. This result cannot represent more middle-aged and older adults with hypertension in China. A more in-depth study is expected to be conducted in the follow-up survey to analyze the influencing factors and related indicators further to improve the study.

## 5. Conclusion

The study showed that SA played a suppression role in the association of PR and DA. DA was at a high level in middle-aged and older adults with hypertension. Medical professionals should improve patients' PR through interventions to reduce DA, increase the likelihood of SA, and promote patient recovery.

## Data availability statement

The raw data supporting the conclusions of this article will be made available by the authors, without undue reservation.

## Ethics statement

All individuals have provided informed consent before the data collection. Approval for this study was given by the Medical Ethics Committee of Wannan Medical College (approval number: 2021-3) and all participants provided informed consent. All methods were performed in accordance with the Declaration of Helsinki. The patients/participants provided their written informed consent to participate in this study.

## Author contributions

LZ: designed the research, supervised the data collection, and collected the data. MW: analyzed the data and drafted the paper. MW, LZ, LG, HL, JM, HS, ZG, and MH: revised the paper. All authors read and approved the final manuscript.

## References

[1] Fang EF Scheibye-Knudsen M Jahn HJ Li J Ling L Guo H. A research agenda for aging in China in the 21st century. Ageing Res Rev. (2015) 24:197–205. 10.1016/j.arr.2015.08.003 26304837 PMC5179143

[2] Sun R Cao H Zhu X Liu JP Dong E. Current aging research in China. Protein Cell. (2015) 6:314–21. 10.1007/s13238-015-0145-5 25779341 PMC4417678

[3] The L. Ageing in China: A ticking bomb. Lancet. (2016) 388:2058. 10.1016/S0140-6736(16)32058-X 27968735

[4] Wang MY Sung HC Liu JY. Population aging and its impact on human wellbeing in China. Front Public Health. (2022) 10:883566. 10.3389/fpubh.2022.883566 35419339 PMC8995787

[5] Lee WJ Peng LN Lin MH Loh CH Chen LK. Determinants and indicators of successful ageing associated with mortality: A 4-year population-based study. Aging. (2020) 12:2670–9. 10.18632/aging.102769 32028266 PMC7041724

[6] Estebsari F Dastoorpoor M Khalifehkandi ZR Nouri A Mostafaei D Hosseini M. The concept of successful aging: A review article. Curr Aging Sci. (2020) 13:4–10. 10.2174/1874609812666191023130117 31657693 PMC7403646

[7] Rowe JW Kahn RL. Successful aging. Gerontologist. (1997) 37:433–40. 10.1093/geront/37.4.433 9279031

[8] Crowther MR Parker MW Achenbaum WA Larimore WL Koenig HG. Rowe and Kahn's model of successful aging revisited: Positive spirituality–the forgotten factor. Gerontologist. (2002) 42:613–20. 10.1093/geront/42.5.613 12351796

[9] Fang EF Xie C Schenkel JA Wu C Long Q Cui H. A research agenda for ageing in China in the 21st century (2nd edition): Focusing on basic and translational research, long-term care, policy and social networks. Ageing Res Rev. (2020) 64:101174. 10.1016/j.arr.2020.101174 32971255 PMC7505078

[10] Wang L Kong L Wu F Bai Y Burton R. Preventing chronic diseases in China. Lancet. (2005) 366:1821–4. 10.1016/S0140-6736(05)67344-8 16298221

[11] GBD 2016 Causes of Death Collaborators. Global, regional, and national age-sex specific mortality for 264 causes of death, 1980-2016: A systematic analysis for the global burden of disease study 2016. Lancet. (2017) 390:1151–210. 10.1016/S0140-6736(17)32152-9 28919116 PMC5605883

[12] Liu J Bu X Wei L Wang X Lai L Dong C. Global burden of cardiovascular diseases attributable to hypertension in young adults from 1990 to 2019. J Hypertens. (2021) 39:2488–96. 10.1097/HJH.0000000000002958 34269332

[13] Turana Y Tengkawan J Chia YC Nathaniel M Wang JG Sukonthasarn A. Hypertension and stroke in Asia: A comprehensive review from hope Asia. J Clin Hypertens. (2021) 23:513–21. 10.1111/jch.14099 33190399 PMC8029540

[14] Olsen MH Angell SY Asma S Boutouyrie P Burger D Chirinos JA. A call to action and a lifecourse strategy to address the global burden of raised blood pressure on current and future generations: The lancet commission on hypertension. Lancet. (2016) 388:2665–712. 10.1016/S0140-6736(16)31134-5 27671667

[15] Mohammadpour A Sadeghmoghadam L Shareinia H Jahani S Amiri F. Investigating the role of perception of aging and associated factors in death anxiety among the elderly. Clin Interv Aging. (2018) 13:405–10. 10.2147/CIA.S150697 29588578 PMC5858545

[16] Douglas DJ. Sociology of death; an analysis of death-related behavior. Am J Nurs. (1972) 72:364–5. 10.1097/00000446-197202000-00050

[17] Tadic M Cuspidi C Grassi G Mancia G. COVID-19 and arterial hypertension: Hypothesis or evidence? J Clin Hypertens. (2020) 22:1120–6. 10.1111/jch.13925 32627330 PMC7362072

[18] Gundogan S Arpaci I. Depression as a mediator between fear of COVID-19 and death anxiety. Curr Psychol. (2022) 22:1–8. 10.1007/s12144-022-03120-z 35496364 PMC9041276

[19] Mirhosseini S Dadgari A Basirinezhad MH Mohammadpourhodki R Ebrahimi H. The proportion of death anxiety and its related factors during the COVID-19 pandemic in the Iranian Population. Fam Med Prim Care Rev. (2021) 23:103154. 10.5114/fmpcr.2021.103154

[20] Beydag KD. Factors affecting the death anxiety levels of relatives of cancer patients undergoing treatment. Asian Pac J Cancer Prev. (2012) 13:2405–8. 10.7314/APJCP.2012.13.5.2405 22901229

[21] Emanuel EJ Fairclough DL Wolfe P Emanuel LL. Talking with terminally ill patients and their caregivers about death, dying, and bereavement: Is it stressful? Is it helpful? Archiv Internal Med. (2004) 164:1999–2004. 10.1001/archinte.164.18.1999 15477434

[22] Iverach L Menzies RG Menzies RE. Death anxiety and its role in psychopathology: Reviewing the status of a transdiagnostic construct. Clin Psychol Rev. (2014) 34:580–93. 10.1016/j.cpr.2014.09.002 25306232

[23] Dadfar M Lester D. Death concern and death obsession in Iranian Nurses. Psychol Rep. (2015) 116:704–9. 10.2466/12.13.PR0.116k30w5 26030202

[24] Gesser G Wong P Reker GT. Death attitudes across the life-span: The development and validation of the death attitude profile (Dap). Omega. (1988) 18:113–28. 10.2190/0DQB-7Q1E-2BER-H6YC

[25] Depaola SJ Griffin M Young JR Neimeyer RA. Death anxiety and attitudes toward the elderly among older adults: The role of gender and ethnicity. Death Stud. (2003) 27:335–54. 10.1080/07481180302904 12749378

[26] Popa-Velea O Diaconescu L Jidveian Popescu M Trutescu C. Resilience and active coping style: Effects on the self-reported quality of life in cancer patients. Int J Psychiatry Med. (2017) 52:124–36. 10.1177/0091217417720895 28792288

[27] Rutter M. Psychosocial resilience and protective mechanisms. Am J Orthopsychiatry. (1987) 57:316–31. 10.1111/j.1939-0025.1987.tb03541.x 3303954

[28] Francalancia J Mavrogiorgou P Juckel G Mitrovic T Kuhle J Naegelin Y. Death anxiety and attitudes towards death in patients with multiple sclerosis: An exploratory study. Brain Sci. (2021) 11:80964. 10.3390/brainsci11080964 34439584 PMC8391402

[29] Hong Y Yuhan L Youhui G Zhanying W Shili Z Xiaoting H. Death anxiety among advanced cancer patients: A cross-sectional survey. Support Care Cancer. (2022) 30:3531–9. 10.1007/s00520-022-06795-z 35018522 PMC8752389

[30] Connor KM Davidson JR. Development of a new resilience scale: The Connor-Davidson Resilience Scale (Cd-Risc). Depr Anxiety. (2003) 18:76–82. 10.1002/da.10113 12964174

[31] Troutman M Nies MA Small S Bates A. The development and testing of an instrument to measure successful aging. Res Gerontol Nurs. (2011) 4:221–32. 10.3928/19404921-20110106-02 21261228

[32] Lee JE Kahana B Kahana E. Successful aging from the viewpoint of older adults: Development of a brief successful aging inventory (Sai). Gerontology. (2017) 63:359–71. 10.1159/000455252 28297704

[33] Templer DI. The construction and validation of a death anxiety scale. J Gen Psychol. (1970) 82:165–77. 10.1080/00221309.1970.9920634 4394812

[34] Yang H Zhang J Lu Y Li M. A Chinese version of a likert-type death anxiety scale for colorectal cancer patients. Int J Nurs Sci. (2016) 3:337–41. 10.1016/j.ijnss.2016.11.002

[35] Kendall B. Multivariate Analysis. Griffin (1975).

[36] Hayes AF. Introduction to Mediation, Moderation, and Conditional Process Analysis: A Regression-Based Approach: Introduction to Mediation, Moderation, and Conditional Process Analysis: A Regression-Based Approach (2013).

[37] Soleimani MA Bahrami N Zarabadi-Pour S Motalebi SA Parker A Chan YH. Predictors of death anxiety among patients with heart disease. Death Stud. (2020) 44:160–7. 10.1080/07481187.2018.1527416 30407129

[38] Tam HL Chung SF Wang Q. Urban-rural disparities in hypertension management among middle-aged and older patients: results of a Chinese National Study. Chronic Illness. (2018) 2022:17423953221102627. 10.1177/17423953221102627 35603631

[39] Chen X Orom H Hay JL Waters EA Schofield E Li Y. Differences in rural and urban health information access and use. J Rural Health. (2019) 35:405–17. 10.1111/jrh.12335 30444935 PMC6522336

[40] Lonetto R Fleming S Mercer GW. The structure of death anxiety: A factor analytic study. J Pers Assess. (1979) 43:388–92. 10.1207/s15327752jpa4304_9 480113

[41] Fortner BV Neimeyer RA. Death anxiety in older adults: A quantitative review. Death Stud. (1999) 23:387–411. 10.1080/074811899200920 10558505

[42] Gonen G Kaymak SU Cankurtaran ES Karslioglu EH Ozalp E Soygur H. The factors contributing to death anxiety in cancer patients. J Psychosoc Oncol. (2012) 30:347–58. 10.1080/07347332.2012.664260 22571248

[43] Mozaffarian D Benjamin EJ Go AS Arnett DK Blaha MJ Cushman M. Heart disease and stroke statistics-2016 update: A report from the American Heart Association. Circulation. (2016) 133:e38–360. 10.1161/CIR.0000000000000350 26673558

[44] Carnethon MR Evans NS Church TS Lewis CE Schreiner PJ Jacobs DR Jr. Joint associations of physical activity and aerobic fitness on the development of incident hypertension: Coronary artery risk development in young adults. Hypertension. (2010) 56:49–55. 10.1161/HYPERTENSIONAHA.109.147603 20516395 PMC2909350

[45] Sun Z Zheng L Detrano R Zhang X Xu C Li J. Incidence and predictors of hypertension among rural Chinese adults: Results from Liaoning Province. Ann Fam Med. (2010) 8:19–24. 10.1370/afm.1018 20065274 PMC2807383

[46] Vina J Sanchis-Gomar F Martinez-Bello V Gomez-Cabrera MC. Exercise acts as a drug; the pharmacological benefits of exercise. Br J Pharmacol. (2012) 167:1–12. 10.1111/j.1476-5381.2012.01970.x 22486393 PMC3448908

[47] Forouzanfar MH Liu P Roth GA Ng M Biryukov S Marczak L. Global burden of hypertension and systolic blood pressure of at least 110 to 115 Mm Hg, 1990-2015. J Am Med Assoc. (2017) 317:165–82. 10.1001/jama.2016.19043 28097354

[48] Sandvik L Erikssen J Thaulow E Erikssen G Mundal R Rodahl K. Physical fitness as a predictor of mortality among healthy, middle-aged Norwegian men. N Engl J Med. (1993) 328:533–7. 10.1056/NEJM199302253280803 8426620

[49] Yusuf S Hawken S Ounpuu S Dans T Avezum A Lanas F. Effect of potentially modifiable risk factors associated with myocardial infarction in 52 countries (the interheart study): Case-control study. Lancet. (2004) 364:937–52. 10.1016/S0140-6736(04)17018-9 15364185

[50] Garber CE Blissmer B Deschenes MR Franklin BA Lamonte MJ Lee IM. Quantity and quality of exercise for developing and maintaining cardiorespiratory, musculoskeletal, and neuromotor fitness in apparently healthy adults: Guidance for prescribing exercise. Med Sci Sports Exer. (2011) 43:1334–59. 10.1249/MSS.0b013e318213fefb 21694556

[51] Moraes-Silva IC Mostarda CT Silva-Filho AC Irigoyen MC. Hypertension and exercise training: Evidence from clinical studies. Adv Exp Med Biol. (2017) 1000:65–84. 10.1007/978-981-10-4304-8_5 29098616

[52] Hasan M Sutradhar I Akter T Das Gupta R Joshi H Haider MR. Prevalence and determinants of hypertension among adult population in Nepal: Data from Nepal Demographic and Health Survey 2016. PLoS ONE. (2018) 13:e0198028. 10.1371/journal.pone.0198028 29852006 PMC5978874

[53] Dimeo F Pagonas N Seibert F Arndt R Zidek W Westhoff TH. Aerobic exercise reduces blood pressure in resistant hypertension. Hypertension. (2012) 60:653–8. 10.1161/HYPERTENSIONAHA.112.197780 22802220

[54] Izadi MR Ghardashi Afousi A Asvadi Fard M Babaee Bigi MA. High-intensity interval training lowers blood pressure and improves apelin and nox plasma levels in older treated hypertensive individuals. J Physiol Biochem. (2018) 74:47–55. 10.1007/s13105-017-0602-0 29214526

[55] Damorim IR Santos TM Barros GWP Carvalho PRC. Kinetics of hypotension during 50 sessions of resistance and aerobic training in hypertensive patients: A randomized clinical trial. Arq Bras Cardiol. (2017) 108:323–30. 10.5935/abc.20170029 28380132 PMC5421471

[56] Li J Yu F Huang N Lu J Xu W Liu N. Effect of baduanjin exercise on patients with chronic heart failure: Protocol for a systematic review and meta-analysis. BMJ Open. (2019) 9:e028771. 10.1136/bmjopen-2018-028771 31350246 PMC6661575

[57] Ma Z Lei H Tian K Liu Z Chen Y Yang H. Baduanjin exercise in the treatment of hypertension: A systematic review and meta-analysis. Front Cardiovas Med. (2022) 9:936018. 10.3389/fcvm.2022.936018 36046185 PMC9421065

[58] Jahnke R Larkey L Rogers C Etnier J Lin F. A comprehensive review of health benefits of Qigong and Tai Chi. Am J Health Promot. (2010) 24:e1–e25. 10.4278/ajhp.081013-LIT-248 20594090 PMC3085832

[59] Li GY Wang W Liu GL Zhang Y. Effects of Tai Chi on balance and gait in stroke survivors: A systematic meta-analysis of randomized controlled trials. J Rehabil Med. (2018) 50:582–8. 10.2340/16501977-2346 29736553

[60] Leung LYL Tam HL Ho JKM. Effectiveness of Tai Chi on older adults: A systematic review of systematic reviews with re-meta-analysis. Arch Gerontol Geriatr. (2022) 103:104796. 10.1016/j.archger.2022.104796 36058045

[61] Wong EML Leung DYP Tam HL Wang Q Yeung KW Leung AYM. The effect of a lifestyle intervention program using a mobile application for adults with metabolic syndrome, versus the effect of a program using a booklet: A pilot randomized controlled trial. Clin Interv Aging. (2021) 16:633–44. 10.2147/CIA.S303920 33888981 PMC8057802

[62] Wong EML Tam HL Leung AYM Cheung ASP Cheung KC Leung DYP. Impacts of educational interventions with support of mobile app versus booklet for patients with hypertension and metabolic syndrome: A secondary data analysis. Int J Environ Res Public Health. (2022) 19:12591. 10.3390/ijerph191912591 36231891 PMC9565212

[63] Yang H Wang Z Yu W Gu Y Shao J Zhang Y. Structural equation model of factors related to death anxiety for Chinese patients with cancer. Omega. (2022) 2022:302228221078349. 10.1177/00302228221078349 35272522

[64] Boškailo E Franjić D Jurić I Kiseljaković E Marijanović I Babić D. Resilience and quality of life of patients with breast cancer. Psychiatr Danub. (2021) 33:572–9. 34718283

[65] Huang Y Huang Y Bao M Zheng S Du T Wu K. Psychological resilience of women after breast cancer surgery: A cross-sectional study of associated influencing factors. Psychol Health Med. (2019) 24:866–78. 10.1080/13548506.2019.1574353 30722683

[66] Rosenberg AR Syrjala KL Martin PJ Flowers ME Carpenter PA Salit RB. Resilience, health, and quality of life among long-term survivors of hematopoietic cell transplantation. Cancer. (2015) 121:4250–7. 10.1002/cncr.29651 26288023 PMC4666727

[67] Qiu C Shao D Yao Y Zhao Y Zang X. Self-management and psychological resilience moderate the relationships between symptoms and health-related quality of life among patients with hypertension in China. Qual Life Res. (2019) 28:2585–95. 10.1007/s11136-019-02191-z 31049824

[68] Vehling S Mehnert-Theuerkauf A Glaesmer H Bokemeyer C Oechsle K Härter M. Thoughts of death and suicidality among patients with cancer: Examining subtypes and their association with mental disorders. Psychooncology. (2021) 30:2023–31. 10.1002/pon.5706 33864314

[69] Kuang D Gu DF Cao H Yuan QF Dong ZX Yu D. Impacts of psychological resilience on self-efficacy and quality of life in patients with diabetic foot ulcers: A prospective cross-sectional study. Ann Palliat Med. (2021) 10:5610–8. 10.21037/apm-21-967 34044569

[70] Ye ZJ Guan HJ Wu LH Xiao MY Luo DM Quan XM. Resilience and psychosocial function among mainland Chinese parents of children with cancer: A cross-sectional survey. Cancer Nurs. (2015) 38:466–74. 10.1097/NCC.0000000000000220 25629894

[71] Hayman KJ Kerse N Consedine NS. Resilience in context: The special case of advanced age. Aging Ment Health. (2017) 21:577–85. 10.1080/13607863.2016.1196336 27333589

[72] Belikov AV. Age-related diseases as vicious cycles. Ageing Res Rev. (2019) 49:11–26. 10.1016/j.arr.2018.11.002 30458244

[73] Sun W Watanabe M Tanimoto Y Shibutani T Kono R Saito M. Factors associated with good self-rated health of non-disabled elderly living alone in Japan: A cross-sectional study. BMC Public Health. (2007) 7:297. 10.1186/1471-2458-7-297 17949511 PMC2186322

[74] Depp CA Jeste DV. Definitions and predictors of successful aging: A comprehensive review of larger quantitative studies. Am J Geriatr Psychiatry. (2006) 14:6–20. 10.1097/01.JGP.0000192501.03069.bc 16407577

[75] Moore RC Eyler LT Mausbach BT Zlatar ZZ Thompson WK Peavy G. Complex interplay between health and successful aging: Role of perceived stress, resilience, and social support. Am J Geriatr Psychiatry. (2015) 23:622–32. 10.1016/j.jagp.2014.08.004 25217186 PMC4329284

[76] Bowling A Iliffe S. Psychological approach to successful ageing predicts future quality of life in older adults. Health Qual Life Outcomes. (2011) 9:13. 10.1186/1477-7525-9-13 21388546 PMC3063186

[77] Spahni S Morselli D Perrig-Chiello P Bennett KM. Patterns of psychological adaptation to spousal bereavement in old age. Gerontology. (2015) 61:456–68. 10.1159/000371444 25720748

[78] Majnarić LT Bosnić Z Guljaš S Vučić D Kurevija T Volarić M. Low psychological resilience in older individuals: An association with increased inflammation, oxidative stress and the presence of chronic medical conditions. Int J Mol Sci. (2021) 22:168970. 10.3390/ijms22168970 34445675 PMC8396457

[79] Ong AD Bergeman CS Bisconti TL Wallace KA. Psychological resilience, positive emotions, and successful adaptation to stress in later life. J Pers Soc Psychol. (2006) 91:730–49. 10.1037/0022-3514.91.4.730 17014296

[80] MacKinnon DP Krull JL Lockwood CM. Equivalence of the mediation, confounding and suppression effect. Prev Sci. (2000) 1:173–81. 10.1023/A:1026595011371 11523746 PMC2819361

[81] Bernabe-Ortiz A Sanchez JF Carrillo-Larco RM Gilman RH Poterico JA Quispe R. Rural-to-urban migration and risk of hypertension: Longitudinal results of the Peru Migrant Study. J Hum Hypertens. (2017) 31:22–8. 10.1038/jhh.2015.124 26865219 PMC4981561

[82] Franklin SS Gustin WT Wong ND Larson MG Weber MA Kannel WB. Hemodynamic patterns of age-related changes in blood pressure: The Framingham Heart Study. Circulation. (1997) 96:308–15. 10.1161/01.CIR.96.1.308 9236450

